# Improving quality of remote mental health consultations during COVID-19

**DOI:** 10.1192/bjo.2021.488

**Published:** 2021-06-18

**Authors:** Phoebe Collins, Karin krall

**Affiliations:** Oxleas NHS Foundation Trust, London

## Abstract

**Aims:**

During the first wave of coronavirus pandemic, many Psychiatry outpatient appointments moved rapidly to remote or ‘virtual’ to protect patients and staff from infection. Telephone consultations do not allow assessment of appearance or other visual aspects of behaviour/affect, yet these are core components of Mental State Examination. Videoconsultation software was unfamiliar to many mental health clinicians, with obstacles including hardware availability, software provision and skills, data security as well as lack of clinican motivation and confidence preventing rapid uptake. I wanted to take advantage of excellent IT support, and NHS England funding of software licence, to drive introduction of Attend Anywhere patient videoconsultation (‘telepsychiatry’) software within my local ADAPT (Anxiety, Depression and Personality Disorder,Trauma) Community Mental Health Team from April 2020 onwards.

**Method:**

I assembled a small group of clinicans to take part in a local pilot of Attend Anywhere software. One Care Coordinator, a Consultant Psychologist, two Consultant Psychiatrists and myself completed satisfaction and confidience scores throughout an 8 week period. Number of videoconsultation outpatient appointments offered to and accepted by patients were also recorded. Weekly group meetings were deemed impossible to schedule given pandemic workloads, so we used 1:1 quick remote catchups, identifying and troubleshooting obstacles, working with IT implement a work-around when the team hit a technical brick wall.

**Result:**

Clinician confidence and satisfaction increased significantly during this period, as did number of offered & completed video consultations.

Attend Anywhere consultations were used for up to 25% of clinician weekly workload.

Clinicians who manage their own diaries started quickly

It was difficult to successfully engage Administration team to organise Attend Anywhere test calls, leading to slow uptake for Consultant Psychiatrists who do not manage their own diaries.

**Conclusion:**

Patient obstacles to use of Attend Anywhere appeared to be idiosyncratic and multifactorial, including poverty, digital exclusion, lack of privacy at home, and clinical history of online grooming. However, some patients already used Attend Anyhwere software with their physical health teams, while others prefer videocall to phone. Age was not an obstacle.

Once this small group of clinicians began to use software successfully, it had a snowball effect within the team and other clinicians asked to sign up for the service. Full support from Administration teams will be crucial to increasing videocalls within the service. Clinicians suggested offering videoconsultation as an opt-out service and requested additional functionality from the software to widen use.

